# Highly Regular Hexagonally-Arranged Nanostructures on Ni-W Alloy Tapes upon Irradiation with Ultrashort UV Laser Pulses

**DOI:** 10.3390/nano12142380

**Published:** 2022-07-12

**Authors:** Luis Porta-Velilla, Neslihan Turan, Álvaro Cubero, Wei Shao, Hongtao Li, Germán F. de la Fuente, Elena Martínez, Ángel Larrea, Miguel Castro, Haluk Koralay, Şükrü Çavdar, Jörn Bonse, Luis A. Angurel

**Affiliations:** 1 Instituto de Nanociencia y Materiales de Aragón (CSIC-University of Zaragoza), C/María de Luna 3, 50018 Zaragoza, Spain; porveli@unizar.es (L.P.-V.); neslihanturan@gazi.edu.tr (N.T.); acubero@unizar.es (Á.C.); wshao@bjut.edu.cn (W.S.); li_ht@163.com (H.L.); german.delafuente.leis@csic.es (G.F.d.l.F.); elenamar@unizar.es (E.M.); alarrea@unizar.es (Á.L.); mcastro@unizar.es (M.C.); 2 Department of Physics, Faculty of Science, Gazi University, Teknikokullar, 06500 Ankara, Turkey; koralay@gazi.edu.tr (H.K.); cavdar@gazi.edu.tr (Ş.Ç.); 3 Institute of Welding and Surface Engineering Technology, Faculty of Materials and Manufacturing, Beijing University of Technology, Beijing 100124, China; 4 College of Materials Science and Engineering, Nanjing Tech University, Nanjing 210009, China; 5 Bundesanstalt für Materialforschung und -prüfung (BAM), Unter den Eichen 87, 12205 Berlin, Germany; joern.bonse@bam.de

**Keywords:** ultrashort laser processing, laser-induced periodic surface structures (LIPSS, ripples), hexagonally-arranged nano-protrusions, second-generation high temperature superconductor technology, electron microscopy, thermal stability analysis

## Abstract

Nickel tungsten alloy tapes (Ni—5 at% W, 10 mm wide, 80 µm thick, biaxially textured) used in second-generation high temperature superconductor (2G-HTS) technology were laser-processed in air with ultraviolet ps-laser pulses (355 nm wavelength, 300 ps pulse duration, 250–800 kHz pulse repetition frequency). By employing optimized surface scan-processing strategies, various laser-generated periodic surface structures were generated on the tapes. Particularly, distinct surface microstructures and nanostructures were formed. These included sub-wavelength-sized highly-regular hexagonally-arranged nano-protrusions, wavelength-sized line-grating-like laser-induced periodic surface structures (LIPSS, ripples), and larger irregular pyramidal microstructures. The induced surface morphology was characterized in depth by electron-based techniques, including scanning electron microscopy (SEM), electron back scatter diffraction (EBSD), cross-sectional transmission electron microscopy (STEM/TEM) and energy dispersive X-ray spectrometry (EDS). The in-depth EBSD crystallographic analyses indicated a significant impact of the material initial grain orientation on the type of surface nanostructure and microstructure formed upon laser irradiation. Special emphasis was laid on high-resolution material analysis of the hexagonally-arranged nano-protrusions. Their formation mechanism is discussed on the basis of the interplay between electromagnetic scattering effects followed by hydrodynamic matter re-organization after the laser exposure. The temperature stability of the hexagonally-arranged nano-protrusion was explored in post-irradiation thermal annealing experiments, in order to qualify their suitability in 2G-HTS fabrication technology with initial steps deposition temperatures in the range of 773–873 K.

## 1. Introduction

Nickel-based tapes are frequently used in second-generation high temperature superconductor (2G-HTS) technology, e.g., as substrates for additional superconductive layer systems. Nickel exhibits a {100}<100> cube texture after heavy cold rolling and adequate annealing [1]. In these conditions, most of the grains are oriented with one of its three mutually perpendicular cubic 4-fold axes (space group $Fm\bar{3}m$) perpendicular to the tape surface, and another one parallel to the rolling direction. The quality of the achieved grain texture depends on the processing conditions and on the Ni impurity grade [2]. Furthermore, alloying also plays a fundamental role in both the generation of the cube texture and the temperature region where this texture is stable [3]. Alloying with refractory metals, such as Mo, W, Nb and Ta, or with Cr, enabled achievement of thermally stable quality texture at high temperatures, up to and above 800 °C [4,5,6,7]. In addition, these Ni alloys feature an increase of mechanical strength and a weakening of the ferromagnetism of pure Ni. In particular, the Curie temperature and the saturation magnetization values reduce as the alloying element content increases [6].

This combination of properties makes nickel alloys excellent candidates for some applications, such as base-metal electrode capacitor fabrication [8], and as metallic substrates for second-generation high temperature superconductors (2G-HTS), following the so-called Rolling Assisted Biaxially Textured Substrate (RaBiTs) method [1,2,4,5,6]. In the latter, these metallic substrates are coated with appropriate buffer layers before the epitaxial deposition of the REBa_2_Cu_3_O_7−x_ (REBCO, with RE being a rare earth or Y) superconducting layer. The optimum alloy content of the substrate is imposed by a balance between the foreseen weakening (or suppression) of Ni ferromagnetism and the observed reduction of both the grain size and fraction of cube-oriented grains, as the alloy content increases. With regard to the buffer layer, CeO_2_ was initially selected with three main objectives, to transfer the biaxial texture of the metallic substrate to the superconducting material, to avoid Ni diffusion into it, and to reduce Ni oxidation during thermal annealing. This last step is performed in a high oxygen partial pressure atmosphere in order to induce formation of the REBCO superconducting phase. Most promising results were initially limited to very thin tapes (less than 100 nm thickness). Later developments with new architectures, such as 100 nm Y_2_O_3_/200 nm YSZ/50 nm CeO_2_ [3], enabled the fabrication of samples with improved superconducting performance, i.e., high values of the superconducting critical current density. Further increase of their critical current values can be achieved by engineering appropriate defect structures in the superconductor in order to improve magnetic vortex pinning [9,10,11].

Pulsed lasers with a wide range of durations have been applied to process material surfaces, allowing the formation of laser-induced periodic surface structures (LIPSS) under certain conditions. Different laser processing parameters, such as laser wavelength, polarization, fluence, or the number of laser pulses, have been found to define the characteristics of the generated nanostructures [12]. Some models have proposed that their origin can be associated with the interference of the incident laser beam and a surface scattered wave that produces inhomogeneous energy absorption [13]. Moreover, it can be expected that near-surface material grains with different crystal orientations can have different planar densities or surface energies, also influencing the interaction of the material with the laser radiation. Several works have investigated how the intrinsic crystal orientation can modify the generated LIPSS in different materials, such as silicon [14], steel alloys [15,16] or nickel [17,18]. The latter were performed on polycrystalline samples with high grain boundary angles.

Initially, laser treatments were carried out analyzing the evolution of LIPSS as a function of the incidence of several pulses at a fixed position (spot) of the sample. The LIPSS-covered areas were thus limited in size. In more recent studies, galvanometer mirror devices have been used to define large-area laser beam scanning strategies in which the spatial overlap between consecutive laser pulses and scanned lines is independently defined and controlled [19]. Depending on the chosen focusing F-theta lens, however, this conventional laser scanning procedure limits the maximum sample area that can be homogeneously processed. This is related to the restrictions in the acceptable local angle of incidence during the laser treatment, or to constraints imposed by the lens aperture and its specific design. In order to overcome these limitations, we have developed a modified laser line scanning mode [20], in which the laser moves repetitively, alternating along a line, while the sample moves continuously in the perpendicular direction. This scalable scanning method can be used to process long samples and is, therefore, compatible with continuous industrial roll-to-roll processing approaches. It minimizes differences in angle of incidence between different spots, thus assuring a homogeneous laser treatment along the entire sample length.

**Figure 1:**
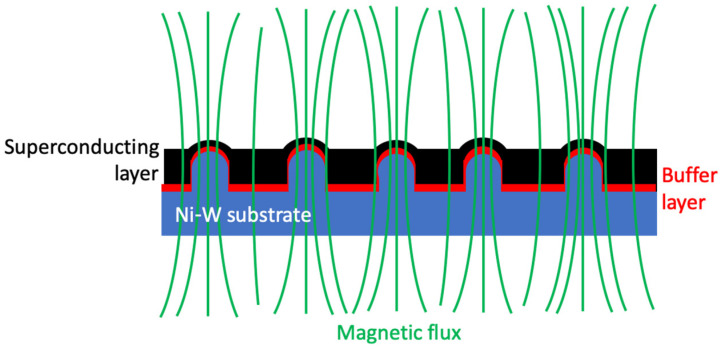
Scheme of a possible way of transferring the laser-generated surface structures in the Ni-W substrate (blue) to the superconducting layer (black) for improving the superconducting properties by pinning the magnetic flux lines (green) at homogeneously distributed sites. The passivating buffer layer (red) prevents chemical reactions between the superconducting layer and the Ni-W substrate.

This work extends our previous studies [21,22] and, in it, we have analyzed the evolution of the surface microstructures and nanostructures generated on a biaxial textured Ni-W alloy tape with an intrinsic high degree of texture, when a 300 ps UV laser was used for irradiation. The main aim was to analyze the effects of the laser processing parameters and crystal grain orientation on the formed microstructure and nanostructure characteristics at the surface, as well as their thermal stability, which is an additional pre-requisite for manufacturing REBCO superconductive tapes. As sketched in Figure 1, the generated surface nanostructures on the substrate could also induce additional nano-sized defects in the superconducting layer associated with these structures. These controlled defects are candidates, under particular experimental conditions, for pinning the so-called “magnetic fluxons” [23], thus improving the superconducting properties.

## 2. Materials and Methods

### 2.1. Laser Processing

A linearly polarized picosecond UV laser (Rofin-Sinar, Germany—wavelength λ= 355 nm, pulse duration *τ*_p_ = 300 ps, and pulse repetition frequencies *f*_rep_ = 250–800 kHz) was used to process the tungsten-alloyed nickel tape surfaces. The beam has an elliptical Gaussian profile with 1/e^2^ intensity decay dimensions [24], at the working distance, 2*a* = 34 μm and 2*b* = 29 μm, along the main axes. Low pulse energy values were applied for the highest pulse repetition frequency value, while higher pulse energies could be used by reducing the frequency.

The laser beam was moved using an optical beam steering system at a given speed, using two different scanning protocols: (i) Beam scanning mode: the sample had a fixed position and the laser scans lines across the surface, controlling the overlapping between successive laser scanning lines. (ii) Line scanning mode: the focused laser spot moved repeatedly in a given direction writing a line of length *l*_L_ with a given scanning speed, *v*_L_, for a given number of times, while the sample was continuously moving in the perpendicular direction at another constant traverse velocity. In this mode, the distance between two laser-line scans was controlled by adjusting the laser scanning speed, the length of the line and the sample traverse velocity. All laser treatments were performed in air.

### 2.2. Characterization Techniques

The surface morphology of the irradiated samples was analyzed in field-emission scanning electron microscopes (SEM, MERLIN Carl Zeiss, Oberkochen, Germany and HITACHI SU5000, Tokyo, Japan) using secondary electron signals, with both in-lens and Everhart-Thornley detectors and electron beam acceleration voltages of 5 kV, unless otherwise indicated. Electron Back Scattering Diffraction (EBSD) experiments were performed with the same microscope using an AztecHKL system from Oxford Instruments (Abingdon, UK) to analyze the surface crystallography.

Specimen cross-section preparation for transmission electron microscopy (TEM) was carried out with a Focused Ion Beam (FIB) in a Dual Beam Helios 650 apparatus of FEI (Lincoln, NE, USA). Prior to the FIB-lamella extraction, samples were carbon-coated for protection. Then, the samples were thinned using 30 kV Ga^+^ ions for the initial steps and 5 kV for final thinning. High-resolution TEM images, energy dispersive X-ray spectrometry (EDS) and electron diffraction were performed in a Tecnai F30 microscope of FEI. High-angle annular dark-field scanning transmission electron microscopy (HAADF-STEM, Tokyo, Japan) was performed with the same microscope for Z-contrast imaging.

### 2.3. Tape Microstructure Characterization

**Figure 2:**
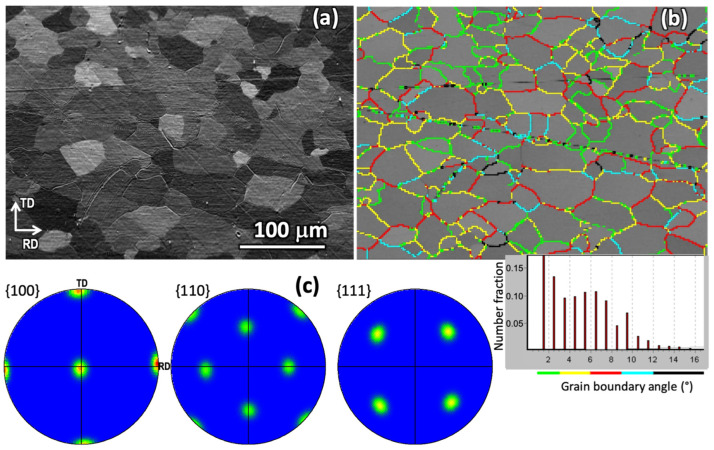
EBSD characterization results of the Ni5W tape before laser irradiation: (**a**) FSD image. (**b**) Grain boundary map of the same sample (identical location and magnification). Colors represent different misorientation angle ranges between adjacent grains as indicated in the histogram below the figure. (**c**) {100}, {110} and {111} pole figures calculated from EBSD measurements of the same sample. RD and TD refer to the rolling direction and transverse direction, respectively.

Laser experiments were performed on commercial 10 mm wide, 80 μm thick polycrystalline Ni-5 at% W (Ni5W) alloy tapes supplied by Evico GmbH (Dresden, Germany), where 5 at% of tungsten were allowed into 95 at% of nickel, prior to a mechanical rolling process for shaping the tape. Studies performed by the supplier revealed that the fraction of cube-oriented grains was well above 99%, as determined by the (100) X-ray reflection intensity [2,4]. Before laser processing, a texture analysis was performed in the sample with EBSD, using a pixel size of 2 × 2 μm^2^ (Figure 2). Figure 2a corresponds to the Forward Scatter Detector (FSD) image of the surface, i.e., perpendicular to the normal direction (ND). The rolling and transverse directions (RD and TD, respectively), are also indicated in the figure. Figure 2b provides the grain boundary map of the same sample using different colors to represent the misorientation angle range between adjacent grains (see corresponding histogram). Figure 2c shows the {100}, {110} and {111} pole figures calculated from EBSD measurements of the same sample using the complete data set. These results confirmed the high cubic texture of the analyzed sample, which also showed low misorientation angles between adjacent grains (Number fraction > 0.91 for angles < 10°).

The optical penetration depth of 355 nm radiation in nickel accounts to 1/α ~ 12 nm, while in tungsten it is just ~7 nm [25,26]. It is reasonable to assume that the optical penetration depth is ruled by the majority element nickel here, and that Ni5W also exhibits an optical penetration depth of the order of 10 nm.

## 3. Definition of Laser Processing Parameters

In order to analyze and correlate the nanostructures and microstructures with the laser processing parameters it is important to consider three different levels: individual laser pulse, pulse-to-pulse overlapping within each individual scanning line (1D scanning configuration) and, finally, extending the line scan to cover a larger area (2D scanning configuration). For this reason, the following processing parameters were defined.

### 3.1. Single Pulse Irradiation

Most of the laser beams have a Gaussian intensity distribution. In our case, the laser had an elliptical profile characterized by a minor *b* and a major *a* ellipse semi-axis (using the 1/e^2^ decay criterium of intensity). In the case of pulsed irradiation, the laser is operated at a given average power, *P*, with pulses emitted at a given repetition frequency, *f*_rep_, and with a given pulse duration, *τ*_p_. The different energy parameters that characterize each pulse are:

Pulse Energy (*E*_p_): The single-pulse energy is calculated as the measured average power (*P*) divided by the pulse repetition frequency (*f*_rep_) during laser operation:

(1) $$E_{p}=\frac{P}{f_{\mathrm{rep}}},$$

Average Pulse Fluence (*F*_av_): Pulse energy per unit area:

(2) $$F_{\mathrm{av}}=\frac{E_{p}}{\pi ab},$$

Irradiance or Intensity (*I*): Average power per unit area of one pulse:

(3) $$I=\frac{E_{p}}{\tau_{p}\;\pi ab}=\frac{F_{\mathrm{av}}}{\tau_{p}},$$

As *τ*_p_ was constant in all the experiments presented in this work, *F*_av_ and *I* were proportional magnitudes. The spatial fluence distribution can be calculated as:

(4) $$F\left(x,y\right)=2\;F_{\mathrm{av}}\exp\left[-2{\left(\frac{x}{a}\right)}^{2}\right]\exp\left[-2{\left(\frac{y}{b}\right)}^{2}\right],$$

Note that the maximum (peak) fluence value at the center of the beam is two times *F*_av_.

### 3.2. Laser Scanning within a Single Line

**Figure 3:**
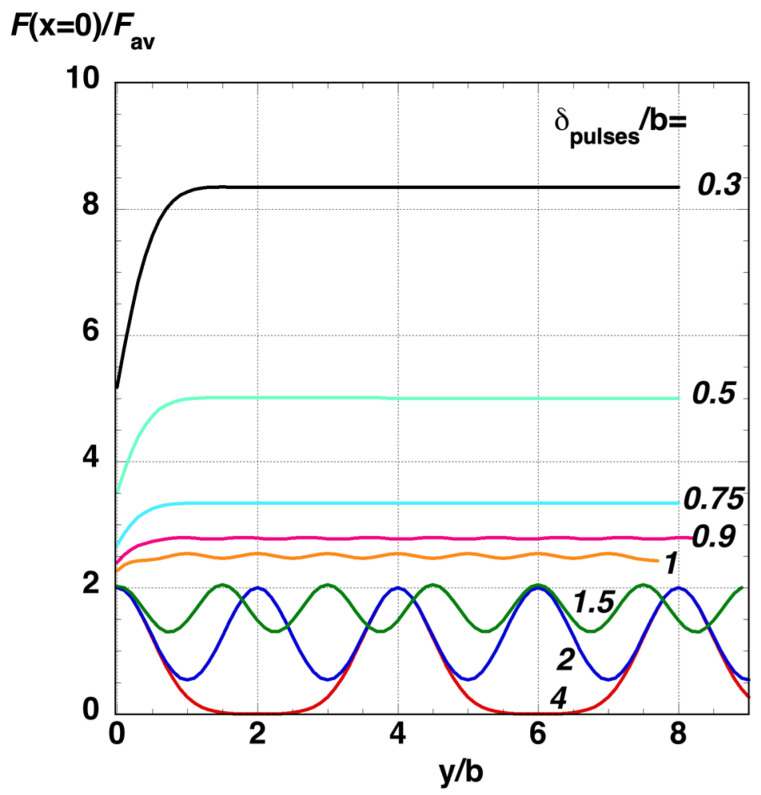
Normalized fluence distribution along a single scan line (*x* = 0) when the laser beam scans in the *y*-direction.

When the laser beam scans one line in the *y*-direction, a series of discrete Gaussian-shaped pulses reaches the sample. The distance between two of these consecutive laser pulses, *δ*_pulses_= *v*_L_/*f*_rep_, is fixed by the ratio of laser scanning speed, *v*_L_, and the repetition frequency, *f*_rep_. Figure 3 shows the normalized fluence distribution [*F*(*x* = 0, *y*/*b*)/*F*_av_] along the *x* = 0 line for different *δ*_pulses_/*b* ratios. When this ratio is sufficiently small (*δ*_pulses_ < 0.9*b*), the fluence distribution can be considered uniform in the *y*-direction, with differences between maxima and minima at *x* = 0 being lower than 1%. In the *x*-direction, a Gaussian distribution is also obtained, given by the expression [27]:

(5) $$F\left(x\right)=F_{\mathrm{center}1D}\exp\left[-2{\left(\frac{x}{a}\right)}^{2}\right]=1.588\;\frac{\pi \;b}{2\;\delta_{\mathrm{pulses}}}\;F_{\mathrm{av}}\exp\left[-2{\left(\frac{x}{a}\right)}^{2}\right],$$

where, *F*_center1D_ is the maximum fluence in the center of the line, *F*(*x* = 0).

Consider *δ*_pulses_/*b* < 0.9 and a time interval Δ*t* >> 1/*f*_rep_. In this period of time, the laser emits *N* = *f*_rep_ Δ*t* pulses and covers a rectangular area described by (2*a*) (*v*_L_ Δ*t*) >> π*ab*. It is possible to define the 1D-accumulated average fluence as:

(6) $$\langle F_{1D}\rangle =\frac{N\;E_{p}}{2\;a\;v_{L}\;\Delta t}=\frac{f_{rep}\;E_{p}}{2\;a\;v_{L}\;}=\frac{f_{rep}\pi \;bE_{p}}{2\;v_{L}\pi ab}=\frac{\pi \;b}{2\;\delta_{\mathrm{pulses}}}\;\frac{E_{p}}{\pi ab}=N_{\mathrm{eff}1D}\;F_{\mathrm{av}},$$

where the effective number of laser pulses per beam spot size is defined in 1D by

(7) $$N_{\mathrm{eff}1D}=\frac{\pi \;b}{2\;\delta_{\mathrm{pulses}}}.$$

### 3.3. 2D Laser Scanning

Finally, when a given area is covered by the focused laser beam, again if the distance between two consecutive scanning lines, *δ*_lines_, is small enough, *δ*_lines_ < 0.9 *a*, the 2D-accumulated average fluence <*F*_2D_> is uniform. The total number of pulses required to cover an area given by *Area* =$\;N_{1}\delta_{\mathrm{pulses}}N_{2}\delta_{\mathrm{lines}}$ is $N_{1}N_{2}$, and <*F*_2D_> can be then calculated as

(8) $$\langle F_{2D}\rangle =\frac{N_{1}N_{2}E_{p}}{Area}=\frac{N_{1}N_{2}E_{p}}{N_{1}\delta_{\mathrm{pulses}}N_{2}\delta_{\mathrm{lines}}}=\frac{\pi ab}{\delta_{\mathrm{pulses}}\delta_{\mathrm{lines}}}\frac{E_{p}}{\pi ab}=N_{\mathrm{eff}2D}\;F_{\mathrm{av}},$$

where the effective number of laser pulses in 2D is defined by

(9) $$N_{\mathrm{eff}2D}=\frac{\pi \;ab}{\delta_{\mathrm{pulses}}\delta_{\mathrm{lines}}}\;.$$

## 4. Results and Discussion

### 4.1. Series of Nanostructures and Microstructures and Their Evolution 

The resulting laser-processed surface topographies were associated with the combined effect of the individual laser pulse hitting a specific spot, the pulse-to-pulse spot overlap within the initial line scanning direction, and the overlapping in the perpendicular direction. It is important to have in mind that the individual time scales of processing were different in each step. In the case of the individual laser pulse, the optical absorption occurred within the pulse duration *τ*_p_, while during the line scan another characteristic time scale was defined by 1/*f*_rep_, as the time between the impact of laser pulses within two adjacent irradiation spots. Moreover, the time that governed the irradiation delay between two adjacent scanned lines was defined by *l*_L_/*v*_L_, where *l*_L_ was the length scanned by the laser beam. In addition, the effective surface absorption could be affected by the surface topography and the number and distribution of defects induced in each of the individual steps, an effect described as incubation [28,29,30]. For this reason, the final generated microstructures and nanostructures would rely on the complete sequence of laser and process parameters and not just on a single one.

#### 4.1.1. Nanostructures Generated in 1D (line) Laser Scanning Treatments

**Figure 4:**
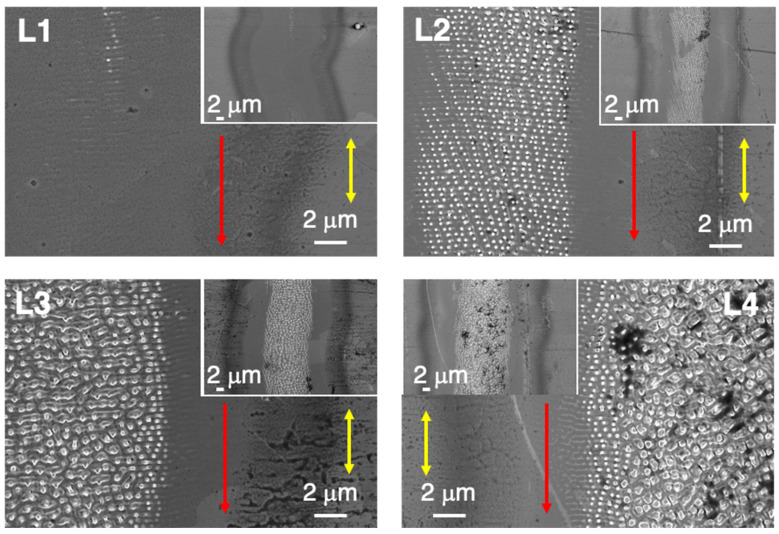
Top-view SEM micrographs (in-lens detector) of nanostructures generated on Ni5W tape in four vertical laser-processed lines obtained by combinations of two values of *F*_av_ and *v*_L_ indicated in Table 1. The insets show the aspect of the complete line section. Red (single) arrows indicate the scanning direction, while the yellow (double) ones indicate the corresponding orientation of the linear laser beam polarization.

Initial laser experiments in the Ni5W tapes were performed processing isolated lines. Figure 4 shows the nanostructures generated at the tape surface after irradiating single lines with the four different combinations of *F*_av_ and <*F*_1D_> that are presented in Table 1. Figure 4 (L1), shows the line generated with individual pulses of *F*_av_ = 43.6 mJ/cm^2^ (*I* = 0.15 GW/cm^2^), a laser scanning speed of *v*_L_ = 100 mm/s, a pulse repetition frequency of *f*_rep_ = 800 kHz and with *N*_eff1D_ = 182.2 and *F*_center1D_ = 12.6 J/cm^2^. With these processing parameters, the modifications induced in the surface were very subtle, observing the onset of LIPSS formation, perpendicular to the scanning direction and a period very close to the laser wavelength. Thus, the LIPSS could be associated with so-called low spatial frequency LIPSS (LSFL) that were caused by an electromagnetic scattering effect [13].

When the scanning speed was reduced to 50 mm/s, both *N*_eff1D_ and *F*_center1D_ doubled with respect to the parameters of line L1 (Figure 4 (L2)). In this case, a nanostructure based on characteristic nano-protrusions within the LIPSS structure appeared in the central part of the line, exhibiting a width of approximately 10 μm. These nano-protrusions formed ordered, dot-like morphology domains belonging to different ripple structures.

Figure 4 (L3) shows the line generated by laser pulses when the applied intensity was 1.6 times higher than in the previous cases, i.e., *F*_av_ = 69.4 mJ/cm^2^. Taking into account that in L2 *v*_L_ reduced to one half in comparison with sample L3, the resulting <*F*_1D_> and *F*_center1D_ values in sample L3 were approximately 80% of those used in L2. A comparison of the nanostructures generated in samples L2 and L3, suggested that the surface-induced modifications were more intense in L3, in spite of the fact that <*F*_1D_> and *F*_center1D_ were both lower for this sample. This implied the relevance of the irradiance and fluence values of each pulse, which were higher for sample L3. In this case, the generated surface structures at the center of the line were micro-protrusions exhibiting a certain degree of order in the direction perpendicular to the beam scan. Finally, Figure 4 (L4) reveals the strongest laser-induced modifications among the four cases shown, with a more disordered micro-protrusion structure than for L3. This could be expected, as it corresponds to the same *F*_av_ value as in L3, but with *v*_L_ reduced to one half. As a conclusion of all these experiments, we deduce that these laser-generated nanostructures and microstructures did not only depend on *F*_av_ nor on <*F*_1D_>, but also the laser processing sequence was determinant.

**Figure 5:**
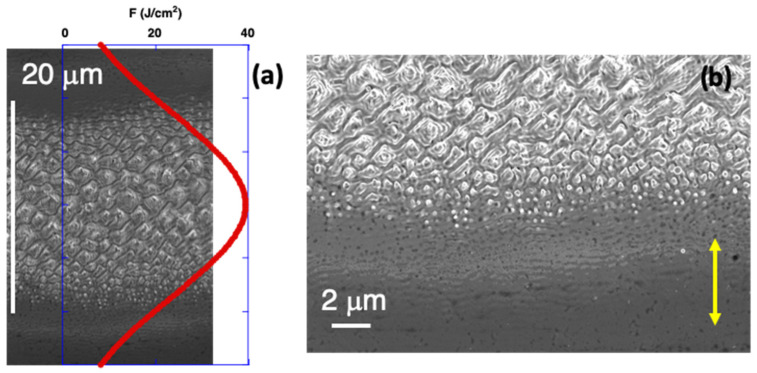
(**a**) Top-view SEM (in-lens) micrograph on the spatial evolution of various nanostructures and microstructures generated on Ni5W tape in a horizontal laser processed line, due to the Gaussian fluence distribution (*F*, red curve). Laser processing parameters were: *F*_av_ = 135.6 mJ/cm^2^, *v*_L_ = 100 mm/s, *f*_rep_ = 800 kHz and <*F*_1D_> = 29.0 J/cm^2^. (**b**) Details of the lower part of the laser processed line. The vertical yellow double-arrow indicates the laser beam polarization direction.

The Gaussian distribution of the fluence in the direction perpendicular to the scanned lines provided information on how the different nanostructures evolved. Figure 5 presents a detail of how these nanostructures were obtained in different regions of a line, generated with the following laser processing parameters: pulses with *F*_av_ = 135.6 mJ/cm^2^ or *I* = 0.45 GW/cm^2^ and *f*_rep_ = 800 kHz; scanning speed *v*_L_ = 100 mm/s, leading to <*F*_1D_> = 29.0 J/cm^2^. In Figure 5a, the fluence profile obtained using Equation (5) is also presented. The nanostructures formed at the lowest fluences, i.e., located at the external border of the line (bottom part in Figure 5b), corresponded to a series of LSFL-LIPSS oriented in the direction perpendicular to the laser beam polarization. As the local fluence increased, some additional nano-protrusions appeared in the top part of the individual LIPSS ridges. A further increase produced some structures that combined nano-protrusions of different sizes (middle part). Finally, characteristic spike-like micro-protrusions, with pyramidal shapes, were seen at the highest local fluence values (top part in Figure 5b).

#### 4.1.2. Surface Structures Generated in 2D (Area) Laser Scanning Treatments

**Figure 6:**
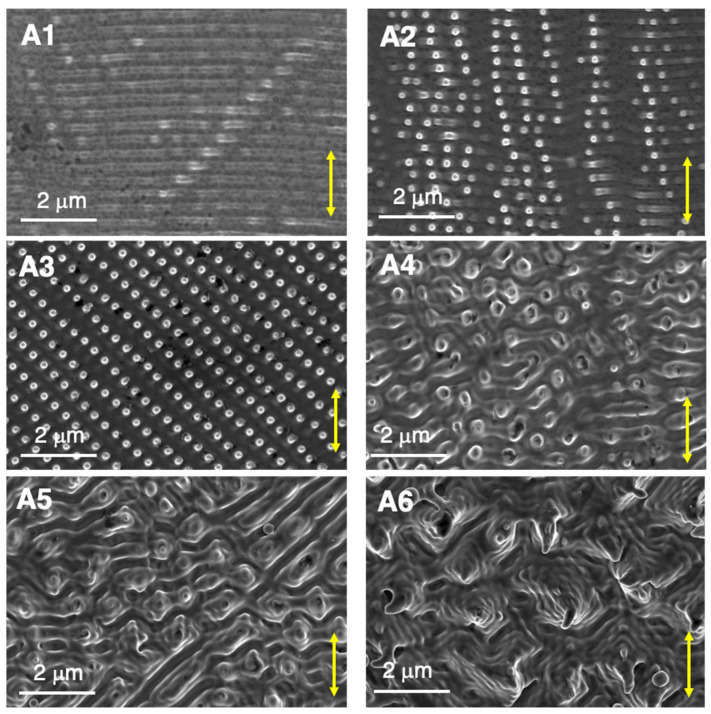
Top-view SEM (in-lens) micrographs of nanostructures and microstructures generated on Ni5W tape using low *F*_av_ and <*F*_1D_> fluence values, as explained in the text and in Table 2. The yellow double-arrows indicate the linear laser beam polarization direction.

In order to analyze the effect of laser irradiation on the formation of self-organized nanostructures and microstructures on the surface of the sample, 2D (area) laser processing had to be considered. Figure 6 shows six types of surface structures (labeled A1–A6) that were formed with low fluence values. The corresponding laser processing parameters are compiled in Table 2.

When *F*_av_ < 56 mJ/cm^2^, <*F*_1D_> < 2 J/cm^2^ and <*F*_2D_> < 17 J/cm^2^, the laser irradiation treatment did not generate any change on the sample surface. When the laser parameters employed for *F*_av_, <*F*_1D_> or <*F*_2D_> were above these threshold values, nanostructures like those labeled as A1 or A2 in Figure 6 started to appear on selected grains of the intrinsic material structure. Some grains exhibited a structure A1 formed by characteristic LSFL. More precisely, they were referred to as type LSFL-I [13], usually observed in strong absorbing materials, such as metals. They were generated in a direction perpendicular to the laser beam polarization and with a period very close to the laser wavelength, in this case, approximately, 355 ± 5 nm. The origin of these LSFL-I structures lay in the electromagnetic scattering and excitation of *Surface Plasmon Polaritons* (SPP) at the material surface and the intra-pulse interference of the associated electromagnetic fields with the incident laser radiation, finally leading to spatially modulated material removal (surface ablation) [13]. A prerequisite of SPP excitation is a constraint on the materials dielectric permittivity *ε*, where the condition Re(*ε*) < −1 should be fulfilled [31]. Since both nickel and tungsten are plasmonically active at 355 nm wavelength [25,26], and just 5% of tungsten is alloyed within nickel, it is reasonable to assume here that the Ni5W tape material is plasmonically active at the laser wavelength.

The original boundary between two grains could be identified in this case as a bright appearing tilted stripe-like feature crossing the horizontal LIPSS ridges, although generally there were no significant changes in the LIPSS structure across this boundary. In addition, it was observed that the LSFL-I orientation could be different between grains, as will be shown in Section 4.3.

In the surface structures labeled as A2, some additional dot-like nano-protrusions appeared on top of the horizontal LIPSS. At the same time, some vertical linearly ordered (chain-like) structures between nano-protrusions that belonged to different LIPSS ridges started to emerge. These nano-protrusions exhibited a highly regular self-ordered 2D structure (labeled as A3) in a number of grains, as the average fluence of the laser treatment increased. This A3 structure was characterized by very regular dot-like surface nano-protrusions that were arranged on parallel sets of lines, crossing at an angle of ~75°. Finally, A4, A5, and A6 structure types showed evolution towards a pyramidal-like microstructure that was obtained with increased values of *F*_av_ ~ 130 mJ/cm^2^, <*F*_1D_> ~ 10 J/cm^2^ and <*F*_2D_> ~ 85 J/cm^2^.

When a microstructure like A6 formed, the amount of molten material was sufficient to obtain a uniform structure across the complete surface. Grain boundaries could not be identified along the surface. By contrast, with lower fluence values, not all the grains were equally affected by laser irradiation, particularly at fluence values close to the threshold. The higher the fluence values were, the larger the number of grains covered by pronounced nanostructures (A2–A3) or microstructures (A4–A5). Nevertheless, it is important to underline that each type of surface structure could be obtained by different combinations of *F*_av_, <*F*_1D_> and <*F*_2D_> values, although generally <*F*_2D_> had less influence on the generated surface structures than *F*_av_ and <*F*_1D_>.

**Figure 7:**
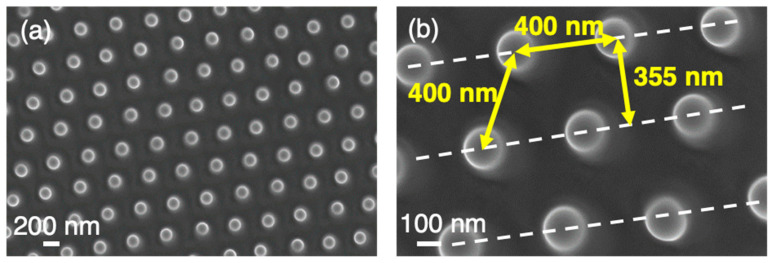
(**a**) Top-view SEM (in-lens) micrographs of hexagonal-lattice self-ordered nanostructures on Ni5W tape. (**b**) Detail of the structure with main dimensions. The dashed white lines indicate the orientation of the original LSFL-I type ripples. The yellow double-arrows indicate characteristic separation distances.

On the other hand, dot-like self-ordered nano-protrusion structures of ~200 nm diameter and arranged in a hexagonal surface-pattern might also be observed on some grains (see Figure 7). This characteristic morphology was obtained with slightly stronger laser irradiation conditions than for those ordered in square surface-lattices (type A3). The distance between adjacent nano-protrusions was about 400 ± 10 nm. As seen in Figure 7b, nano-protrusions generated on each individual LIPSS ridge were able to reach an ordered structure with the nano-protrusions generated in the adjacent LIPSS-ridge, still arranged at distances of approximately 355 ± 5 nm, i.e., the LSFL-I spatial period Ʌ. Such square and hexagonally arranged self-ordered structures have also been reported on single crystal W surfaces after irradiation with an 8 ns Q-switched Nd:YAG laser at 532 and 1064 nm wavelength [32,33] and on Ge using a 120-fs 800 nm wavelength laser [34]. In all the cases, experiments have been performed at controlled atmosphere and cubic and hexagonal lattices were also observed with an array spacing slightly higher than the laser wavelength and only with an angle of incidence very close to the normal to the surface. Furthermore, 2D ordered nanocavities have been also obtained in Ni single crystals with (100) orientation irradiating the sample with ultrashort polarization-crossed sequences of double pulses using a 25 fs Ti:Sapphire laser [17,35].

**Figure 8:**
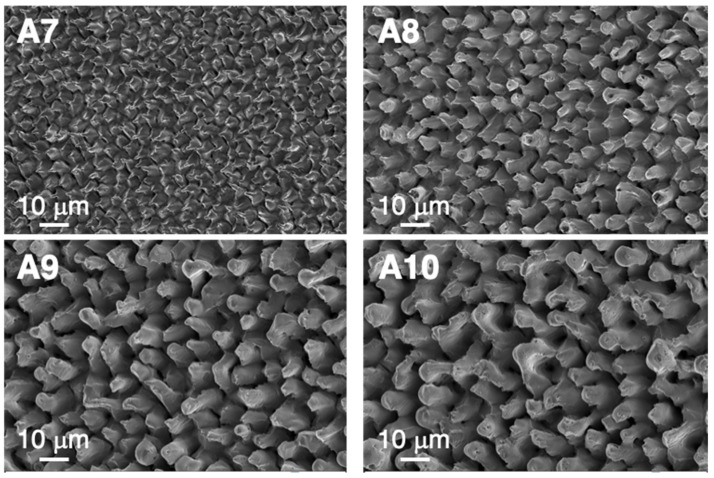
Top-view SEM (SE) images of the laser-processed microstructures generated an the Ni5W tape surface by using high *F*_av_ and <*F*_1D_> values, as explained in the text and in Table 2.

As the laser treatment severity increased, heavier damage emerged in the sample surface, as shown in Figure 8. The micro-scaled surface morphology of sample A7 was similar to that obtained in sample A6, but with larger formed structures, which were around 3–5 μm in lateral size. Structures A8, A9 and A10 corresponded to the highest *F*_av_ and <*F*_1D_> values of this series (in the range of 450–700 mJ/cm^2^ and 12–25 J/cm^2^, respectively), leading to larger and deeper surface structures. With these laser conditions, the microstructure appeared uniform in the complete surface and the influence of the metal grain orientation completely disappeared. Similar conical structures have been observed in Ni surfaces after being irradiated with fs lasers [36,37]. Given their irregular spike-like morphology, such laser-structured surfaces exhibit an extremely low surface reflectivity in the visible and near-infrared spectral range and are, therefore, referred to as “black metals” [38].

### 4.2. Cross-Sectional Analysis of the Laser-Induced near-Surface Material Modifications

**Figure 9:**
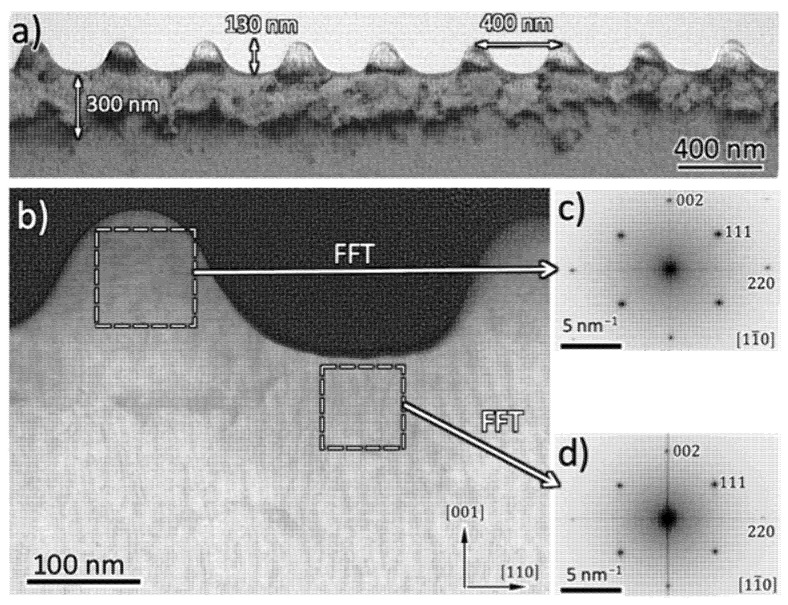
(**a**) TEM brightfield image of a cross-section of a sample showing type A3 nanostructures on a Ni5W tape after laser irradiation. (**b**) Detail of the formed nano-protrusions of the same cross-section using the HAADF-STEM detector. The 2D Fast Fourier Transforms (2D-FFT) of high-resolution images obtained in the different zones indicated by two squares in (**b**) are shown in (**c**) and (**d**), respectively. The bright region in (**a**) corresponds to the deposited carbon protection layer for the TEM cross-section specimen preparation.

In order to obtain additional information about the changes of the intrinsic material structure induced by the laser irradiation at the surface of the samples, cross-sections were investigated using transmission electron microscopy. Figure 9 shows the cross-sectional profile of a Ni5W sample with a type-A3 nanostructure. Very good homogeneity of the formed nano-protrusions, both in size and in lateral separation, was observed, as is clearly seen in Figure 9a. The distance between adjacent nano-protrusions, in the cross-sectional direction used to fabricate the FIB lamella, was approximately 400 nm, which was close to the laser wavelength, and their height was about 130 nm. It is noted that a region of about 100 to 300 nm thickness, just below the formed nano-protrusions was also affected during the laser-processing. Its extent was significantly larger than the optical penetration depth of the UV laser radiation that accounts to 1/α ~ 12.1 nm only at 355 nm wavelength (with α = 8.23 × 10^5^ cm^−1^ being the linear absorption coefficient of nickel taken from [25]).

However, a modified depth of a few hundreds of nanometers was fully in line with the typical extent of the so-called *heat-affected zone* (HAZ) found after scan-processing of solids by ultrashort laser pulses [39]. This was further supported by an estimation of the *thermal diffusion length L*_th_ =$\;\sqrt{2\cdot D\cdot \tau_{p}}$ for single laser pulse irradiation. With the thermal diffusivity *D* = 0.24 cm^2^/s [40] for solid nickel and the pulse duration *τ*_p_ = 300 ps, a value of *L*_th_ ~ 120 nm was obtained here. In such a scenario, the laser radiation was absorbed by an approximately 10 nm thin skin layer at the metal surface, while the dissipation of the deposited optical energy and the resulting material modifications extended at least over a depth *L*_th_ due to the subsequent energy relaxation processes (electron-phonon relaxation, heat diffusion, etc.).

A detail of two neighbored nano-protrusions of the same cross-section using the HAADF-STEM detector is shown in Figure 9b. High-resolution images along the $\left[1\bar{1}0\right]$ zone axis were obtained from the two different regions around the nano-protrusions indicated in Figure 9b. Figure 9c,d show their corresponding 2D Fast Fourier Transforms (2D-FFT). They revealed a high degree of crystallinity in both regions. In addition, the selected area electron diffraction (SAED) patterns obtained from larger areas (~1 µm in diameter) of this region showed the same degree of crystallinity. The dark spots in Figure 9c,d corresponded to electron diffraction at the {220}, {111} and {002} crystallographic lattice planes. It was clearly observed that the nano-protrusions were formed perpendicular to the surface, i.e., along the <001> direction. The difference between both marked areas in Figure 9b was that in the nano-protrusion valley, the lattice parameter determined from the Fast 2D Fourier Transform (2D-FFT) was ~3% larger than at the nano-protrusion itself (compare Figure 9c,d)). Sungurov and Finkel [41] measured a linear dependence between the lattice parameters and the W content in Ni-W alloys. In consequence, this result seems to indicate that the W content is higher in the valley than in the nano-protrusion. In good agreement, EDS line profiles showed that the W content increased from the top part of the nano-protrusion to the inner part (see Figures S1 and S2 in the Supplementary Material). In consequence, it was observed that the crystallographic order was maintained, despite the observed redistribution of W, although it was lower in the nano-protrusions than in the valleys between them.

**Figure 10:**
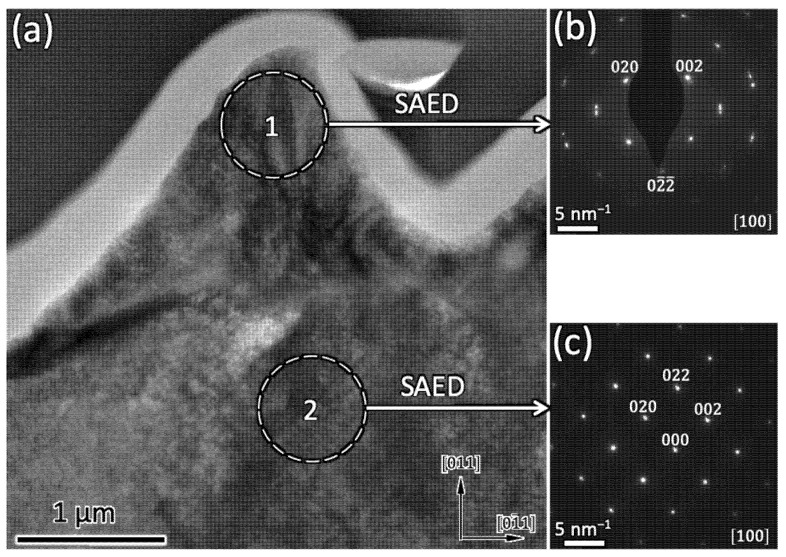
(**a**) Brightfield TEM cross-section of the type A6 microstructures and selected area electron diffraction (SAED) patterns from region 1, in the apex of the protrusion (**b**), and in region 2, just below the protrusion (**c**). The bright layer in (**a**) corresponds to the deposited carbon protection layer for the TEM cross-section specimen preparation.

A similar cross-sectional (S)TEM study was performed for samples characterized by pyramidal-like A6-type structures (Figure 10). The cross-section demonstrated that these kinds of surface structures correspond to resolidified material. In fact, the crystallographic orientation of this region was different from that of the original Ni5W tape. In addition, the mosaic character of the material structure displayed in Figure 10a was significantly higher in the protrusion (region 1 in Figure 10) than in the less-affected material, just below the protrusion (region 2). The associated SAED patterns further support this observation, since the discrete electron diffraction peaks associated with the body of the micro-protrusion tended to broaden, split and smear out in the tangential direction (compare Figure 10b,c). Note that the height of these micro-protrusions was of the order of 1.5 μm, i.e., one order of magnitude larger than for type A3 nano-protrusions.

### 4.3. Influence of Grain Orientation on the Laser-Generated Nanostructures

**Figure 11:**
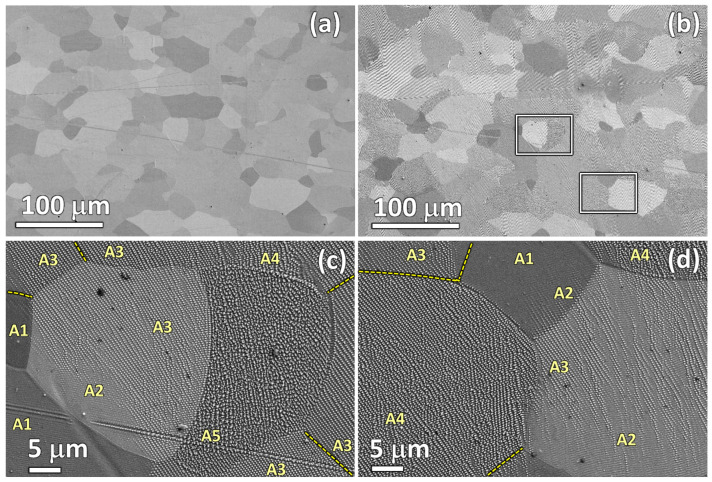
(**a**,**b**) Top-view SEM (SE) micrographs of the analyzed tape, before and after laser irradiation, respectively. Laser irradiation parameters are given in the text. (**c**,**d**) SEM (SE) micrographs showing a detail of the nano- and microstructures generated in the two selected areas highlighted in (**b**) with rectangles. The structure types (A1…A5) are indicated. Discontinuous (yellow) lines correspond to grain boundaries observed with EBSD maps, but not clearly visible in SEM images.

Figure 11a,b show overview SEM images of the identical tape surface area (400 μm × 270 μm), of the as-received and laser processed, respectively. Note that this is the same sample/area previously analyzed with EBSD (Figure 2) prior to the laser processing. It is worth remembering here that the sample was tilted 70° for the latter. For this study, the Ni5W tape was irradiated using the laser line scanning configuration and the processing parameters detailed in Table 3.

As the focusing lens was a F-Theta one and the processing line was not placed at the center of the laser processing area, the laser incidence angle was 82° and the laser scanning direction was parallel to the laser linear polarization axis here.

Figure 11c,d show two representative areas of the tape surface after irradiation and show, in more detail, the generated structure-types (A1 to A5). The exact positions of these zones are highlighted by two white rectangles in Figure 11b. As previously mentioned, these surface nanostructures varied among grains, despite the small differences in grain orientation that were observed in the sample surface (Figure 2). Moreover, for a given grain, the generated structure in the adjacent grains also exerted some influence on the observed structure type near the boundary. This happened in both directions, i.e., either producing an increase or a decrease of laser effects, depending on the type of surface structure formed in the adjacent grain. As an example of this feature, we could observe that the predominant structures formed on the two largest grains at the center of the image in Figure 11c were of types A5 (right large grain) and A3 (left large grain). Nevertheless, in the latter, the left-lower part of the grain exhibited a type A2 structure (even type A1 was observed in the region close to the adjacent grain with an A1 nanostructure). It is also worth noting that, in some cases, the type of laser-generated nanostructure did not vary across certain grain boundaries. This might remain unnoticed in SEM images (see yellow dashed lines in the figure), but is clearly seen through EBSD maps. This effect was also observed, as in Figure 6 (A1).

These observations indicate that the grain orientations of the as-received Ni5W tapes have some influence on the type of structure generated by the laser treatment, particularly in the low fluence regime, very close to the ablation threshold of the irradiated material. On the one hand, this might be caused by differences in the optical absorption processes (grain orientation dependent), leading to differences in the efficiency of the excitation of optical surface scattering effects and defect-mediated excitation of surface electromagnetic waves (SEW) [13]. On the other hand, grain boundaries might also re-scatter such surface waves and impede, or support, the propagation of the SEW across the corresponding grain boundary. Nevertheless, both point towards the involvement of grain-dependent electromagnetic effects as the seed of the laser-generated nanostructures.

**Figure 12:**
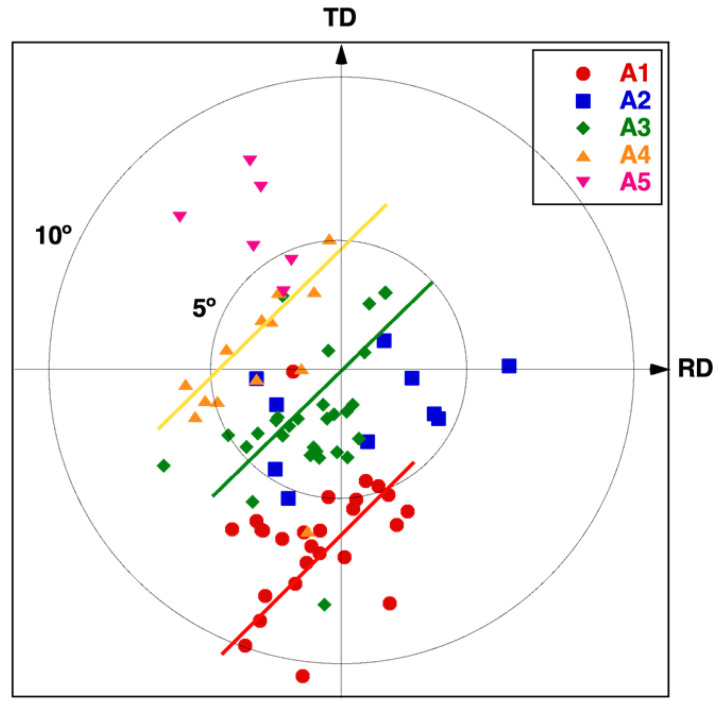
Correlation between the grain orientation before laser irradiation and the laser-generated nanostructures and microstructures (types A1 to A5) on Ni5W tape. Each point represents a grain. Its position in the plot is given by the stereographic projection of the grain orientation prior to laser irradiation, whereas the used symbol corresponds to the laser-generated structure (type A1 to A5) in this particular grain (see further explanation in the text and in Figure S3). RD and TD refer to the rolling direction and transversal direction, respectively. Circumferences indicate specific misorientation angles of 5° and 10° with respect to the surface normal direction (ND, i.e., 0° represented by the origin). The straight lines guide the eye and correspond to preferential orientation trends of grains where different type of structures (A4, A3 or A1 (from top to bottom)) are developed.

It is important, therefore, to further analyze this effect and to establish a correlation between the initial grain orientation of the material surface and the resulting nanostructures for a given set of laser processing parameters. With this aim in mind, the crystallographic orientation in the as-received sample (Figure 2 and Figure 11a) was identified by EBSD for each grain confined to the analyzed surface and, then, associated with the type of nanostructure (A1–A5) generated by the subsequent laser processing (Figure 11b). The results of this procedure are visualized Figure 12, which represents the stereographic projection of one of the main cubic axes of each grain onto the sample’s surface, the closest to the normal direction (for details, see Figure S3 in the Supplementary Material). Each point in the graph, thus, represents a different grain, showing its orientation and the type of nanostructure that was generated on its surface. With this criterion, if a grain was oriented with some of its axes exactly perpendicular to the sample surface, i.e., parallel to the surface normal direction (ND), the corresponding symbol would be placed at the center of the graph. Similarly, the circles in the figure indicated 5° and 10° misorientation values with respect to ND.

The observed grain orientation patterns projected for the A1 to A5 type surface nano- and microstructures were not concentric, indicating that the misorientation with respect to ND was not the main (or not the only) factor determining the generated microstructure. In fact, the rolling direction during manufacturing of the Ni5W tapes prior to the laser irradiation also played a significant role. Some distinct preferential grain orientation trends could, however, be observed for each nanostructure and microstructure type in the figure. Note that each type was grouped in bands (areas around the solid straight lines in the figure), which all ran nearly parallel to the diagonal of the first and third quadrants. Rectangular and hexagonal self-ordered nanostructures (labeled as type A3, green line) were obtained on grains whose orientation was close to this diagonal. The grains associated with the lowest laser-interaction strength (A1, red line) were arranged in separate bands placed below the diagonal, whereas those associated with stronger laser-interaction strength (A4 and A5) were above the diagonal. While the data points of the type A4 surface structures still followed a linear trend (yellow line), this relation became widely lost for the type A5 structures (pink data points). It was also observed that just a few grains did not follow this general behavior. Further analysis revealed that these exceptions corresponded to small-size grains, the nanostructures of which were strongly affected by those of their surrounding grains. 

In view of our EBSD analyzes, presented in Figure 12, and taking into account that EBSD maps were recorded before laser processing, one could summarize that, for laser irradiation conditions close to the materials ablation threshold, small grain crystallographic misorientations could cause important differences in the energy absorbed by the material, finally producing a broad range of nanostructures and microstructures. It should be underlined again that when the laser treatment generated surface microstructures, like those classified as type A6, the resolidified material changed its near-surface crystallographic orientation (compared to the one of the materials initial grain structure).

### 4.4. Generation Mechanisms of Hexagonally-Arranged Nanostructures

While it has already been discussed that grain-dependent electromagnetic absorption and scattering processes are involved in the formation of low fluence type A1–A5 surface nanostructures, another important general aspect in LIPSS formation should be discussed here. This aspect is related to laser-induced hydrodynamic effects that transiently act on the laser-induced surface melt layer.

The observed hexagonal arrangement of the nano-protrusions might also point toward hydrodynamic effects causing such characteristic melt displacement immediately after laser pulse irradiation. Potential scenarios might involve Bénard-Marangoni or other convective instabilities, de-wetting in the thin melt layer, or Rayleigh-Taylor instabilities [42]. For further exploration of the present situation, it is instructive to analyze, in more detail, the optical absorption, and subsequent hydrodynamic evolution, of the ultrashort laser pulse melted metallic nickel surface.

Rudenko et al. generally analyzed, through Finite-Difference Time-Domain (FDTD) calculations, the optical absorption and energy deposition at metallic surfaces that feature nano-bumps and nanoholes [43]. They showed that nano-bumps (such as our nano-protrusions) with radii *R* < *λ*/(2π) on metals exhibited a locally reduced surface absorption in comparison with the plane surface surrounding the nano-bumps. The absorption depth is essentially given through the optical skin depth. Although the condition on *R* was not strictly fulfilled in our case (*R* ~ 200 nm, *λ* = 355 nm), a moderate decrease of the deposited optical energy at the type A3 nano-protrusions might also be expected in our case. This laterally modulated deposition of the optical energy occurred in Ni at depths of the order of ten nanometers (~1/*α*) during the 300 ps laser pulse, with an electron-phonon relaxation time in the sub-ps scale [44]. Hence, the electrons and the lattice temperature of the nickel sample could already be considered in thermal equilibrium during the 300 ps laser pulse. Ni melts at lattice temperatures exceeding 1727 K, forming a surface melt layer with a thickness of the order of one hundred nanometers (~*L*_th_). This molten Ni layer could exhibit local temperature gradients, initially seeded laterally by the modulated deposited absorption/optical energy, and longitudinally by the energy deposition depth. Since relevant thermophysical properties of the molten material (such as the surface tension *σ* and viscosity *μ*) significantly depend on temperature, some thermocapillary forces might act on the liquid surface layer at the nickel surface, leading to its displacement during the lifetime of the laser-induced melt.

Abou Saleh et al. studied the formation of a plethora of different surface nanostructures on nickel upon temporally distributed optical energy deposition through fs-double pulse experiments, complemented by numerical simulations [35]. The authors reported the formation of hexagonally dome-shaped arranged “nano-pits” similar to the nano-protrusions presented here (although somewhat smaller in diameter and distances).

Using the temperature coefficient of the surface tension of molten Ni *γ* = |d*σ*/d*T*|= ~ 4 × 10^−4^ N/(m K) [45] and assuming a typical longitudinal (depth) gradient of *L* ~ 100 nm ~ *L*_th_ along with a temperature difference of Δ*T*_l_ = 1000 K, a typical Marangoni pressure induced force of *P*_M_ ~ *γ*Δ*T*_l_/*L* = 4 × 10^6^ Pa could be estimated that drove the hotter melt regions in the direction of lower temperatures, i.e., towards the positions of the nano-protrusions. The typical time scale required for the development of such a Marangoni convection instability of a certain cell size Ʌ_M_ could be estimated via τ_M_ ~ µ Ʌ_M_^2^/(4 *L γ*Δ*T*), with the viscosity *µ* = 3 × 10^3^ Pa s [42]. Taking for Ʌ_M_ the nearest-neighbor distance of 400 nm (see Figure 7), the characteristic time accounted to τ_M_ ~190 ps and was shorter than the expected lifetime of the laser-induced melt. Hence, in principle it would allow the longitudinal Marangoni instability to take place for the irradiation conditions herein reported.

To further check the relevance of the longitudinal Marangoni effect, the corresponding dimensionless *Marangoni number Ma* = *γ*Δ*T L*/(*µ D*_l_) should be estimated. With the heat diffusivity *D*_l_ ~ 10^−5^ m^2^/s of liquid Ni, and the above specified thermophysical values, the Marangoni number accounted to *Ma* ~ 1.3 in our case. This value was significantly smaller than the critical value of *Ma*_cr_ between 40 and 80 that is generally assumed to be required to develop the Bénard-Marangoni instability [35].

However, as pointed out by Abou Saleh et al. [35], the melt instability can also occur via *transverse* temperature gradients parallel to the surface, generated by the locally modulated optical absorption of the laser radiation. The nature of such surface patterns is then defined by the dimensionless *Prandtl number Pr* = *C*_i_ *μ*/*k*_i_, with *C*_i_ = 630 J/(kg K) being the heat capacity, and *k*_l_ = 50 W/(m K) being the thermal conductivity for liquid Ni [45]. With all given values, it accounted to *Pr* ≈ 0.04 here. This value is significantly smaller than the critical value of *Pr*_cr_ = 0.25. However, for colder Ni, the viscosity is higher. Hence, the Prandtl number *Pr* might approach and exceed the critical value and could then result in the formation of upwardly directed hexagonal nanostructures. Note that this increase of *µ* further decreases *Ma*, making the longitudinal Marangoni effect even less effective at low melt temperatures. It is, therefore, reasonable that the transverse melt instability occurred preferentially here in the low laser fluence regimes here (being responsible for type A3 nano-protrusions), while at higher laser fluences the residual melt layer transiently featured higher temperatures and longitudinal gradients, in favor of increasing *Ma* and the longitudinal Marangoni melt instability.

### 4.5. Annealing Temperature Stability of the Hexagonally-Arranged Nanostructures

These Ni-W tapes are used as substrates for the fabrication of 2G-HTS superconducting tapes. For this reason, it is important to analyze whether these nanostructures are stable at the temperatures required to deposit the different additional layers that are needed to deposit the superconducting oxide. Those temperatures are typically in the range of 800 to 1000 K. These nanostructures, if thermally stable to the latter temperature range, could generate a set of ordered topographic surface defects that could improve the superconducting properties by controlling the defect landscape. Such surface defect engineering would be beneficial to improve vortex pinning and, in consequence, magnetic and transport properties of the 2G superconducting tapes industrially manufactured.

**Figure 13:**
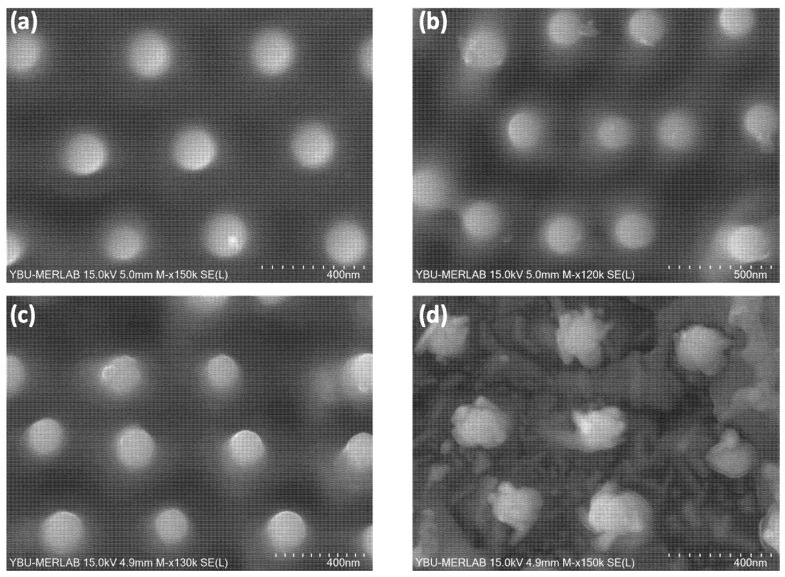
Top-view SEM (SE) images of the modification of the Ni5W tape surface that was laser-textured with type A3 nanostructures after additional thermal annealing treatments of 1 h in Ar atmosphere: Original surface (**a**), annealed at 523 K (**b**), 723 K (**c**) and 973 K (**d**). Electron beam acceleration voltages of 15 kV where used.

In order to investigate the thermal stability of the A3 laser-generated nanostructures, the samples were thermally annealed in a furnace at 523 K, 723 K and 973 K for 60 min in argon atmosphere in order to reduce tape oxidation that takes place at temperatures above 773 K [46]. Figure 13 compares SEM micrographs of an initial tape surface where self-ordered A3 nanostructures were generated and their aspect after the different annealing cycles applied. As a reference, Figure 13a visualizes the laser processed type A3 surface prior to the thermal annealing. In the sample annealed at 523 K (Figure 13b), no significant change was observed within the nanostructures on the surface. Only a leaf-shaped structure started to form on some of the nano-protrusions. Upon increasing the annealing temperature up to 723 K (Figure 13c), these “leaves” increased in size but the nano-protrusion structure was still clearly observed without deterioration. If the annealing temperature was increased to 973 K (Figure 13d), the surface began to flake off and the size of the leaf-like structures on top of the nano-protrusions increased. Nevertheless, even when the contours of the nano-protrusions were weakened, their main features were still visible on the sample surface.

Considering the latter annealing behavior, these nanostructures might be considered thermally stable for their application towards the fabrication of improved 2G-HTS conductors. For instance, Varesi et al. [47] proposed a YBa_2_Cu_3_O_7-x_/CeO_2_/Ni-W architecture. In this fabrication process, an initial 50 nm thick CeO_2_ layer was deposited in a vacuum at 873 K in order to avoid Ni5W tape oxidation, before introducing 10 mTorr of flowing oxygen to reach a final thickness of 700 nm. Subsequently, temperature was slowly increased to 1123 K in order to deposit the superconducting layer. Supposedly, these type A3 nanostructures generated on the Ni5W tape could be transferred to the buffer layer by adjusting the deposition parameters. The stability of these nano-protrusions on the buffer layer should be further analyzed in order to determine the possibility of effectively transferring them to the superconducting layer.

## 5. Conclusions

An in-depth analysis of the evolution of the nanostructures and microstructures generated in a biaxially textured Ni5W tape when its surface was irradiated with a 300 ps UV laser in beam and line scanning configurations was performed. The evolution of the laser-generated surface structures was characterized by high-resolution electron beam-based techniques, including SEM, EDX, EBSD and (S)TEM. Upon increasing the severity of the laser treatment, the induced surface morphology hierarchy followed this sequence: ripples (type LSFL-I), rectangular or hexagonal self-ordered nano-protrusions, and pyramidal microstructures. Different laser processing parameters were introduced to relate the generated nanostructures to the severity of the laser treatment by experimentally controlling the laser pulse energy (fluence), the spatial overlap between pulses during laser scanning, and the overlap between adjacent laser processed lines. It was demonstrated that the laser-generated surface structures could not only be associated to one of the processing parameters. Instead, the complete processing combination that was followed to perform the laser treatment was relevant.

It was found that the nanostructures generated with low laser fluence values strongly depended on the tape alloy crystallographic grain orientation. It was established that, for a given set of laser processing parameters, the generated nanostructures also depend on the original grain crystallographic orientation with respect to the sample surface. An asymmetry between the rolling and the transverse direction was also observed. A specific laser processing regime was identified, where highly regular hexagonally arranged nanodot-like surface protrusions, ~200 nm in diameter, with heights of ~130 nm, and closest-neighbor distances of ~400 nm could be manufactured, while the underlying heat-affected zone was limited to less than 300 nm depth.

High-resolution TEM analyzes of nano-protrusion cross-sections showed that the texture of the tape and the grain crystallinity were maintained during the laser treatment, although some parts of the W alloying element redistributed near the treated surfaces, thus, reducing its content in the top part of the hexagonally-arranged nano-protrusions.

The thermal stability of these laser generated nanostructures was analyzed by thermal annealing experiments, and it was observed that they were stable up to temperatures of the order of 873 K in argon atmosphere. Therefore, the use of this technique for surface nanostructuring of Ni-W substrates opens the possibility of transferring this nanostructure to the buffer layer in 2G-HTS architectures and, from this, to the superconducting layer. This could pave the way to improved superconducting properties of these industrially manufactured tapes, because the laser-generated nanostructures in the Ni-W tape could impose formation of a set of controlled defects in the superconducting layer that could facilitate the pinning of magnetic fluxons and, in consequence, improve the superconducting properties.

## Figures and Tables

**Table 1:** 1D (line) laser processing parameters of the samples presented in Figure 4. The laser was operated at *f*_rep_ = 800 kHz.

Sample	*F*_av_ (mJ/cm^2^)	*v*_L_ (mm/s)	*δ*_pulses_ (μm)	*N*_eff1D_	<*F*_1D_> (J/cm^2^)	*F*_center1D_ (J/cm^2^)
L1	43.6	100	0.13	182.2	7.9	12.6
L2	43.6	50	0.06	364.4	15.9	25.2
L3	69.4	100	0.13	182.2	12.6	20.1
L4	69.4	50	0.06	364.4	25.3	40.2

**Table 2:** 2D (area) laser processing parameters of samples A1 to A10. The laser was operated at *f*_rep_ = 800 kHz for A1–A6 and at 300 kHz for A7–A10, respectively.

Sample	*F*_av_ (mJ/cm^2^)	*v*_L_ (mm/s)	*δ*_pulses_ (μm)	*N*_eff1D_	<*F*_1D_> (J/cm^2^)	*δ*_lines_ (μm)	*N*_eff2D_	<*F*_2D_> (J/cm^2^)
A1	85.5	750	0.94	24.3	2.1	8	103.3	8.8
A2	56.5	750	0.94	24.3	1.4	2	413.0	23.3
A3	69.4	500	0.63	36.4	2.5	2	619.5	43.0
A4	101.7	500	0.63	36.4	3.7	4	309.8	31.5
A5	135.6	500	0.63	36.4	4.9	4	309.8	42.0
A6	135.6	250	0.31	72.9	9.9	4	619.5	84.0
A7	348.7	250	0.83	27.3	9.5	4	232.3	81.0
A8	456.3	250	0.83	27.3	12.5	4	232.3	106.0
A9	563.9	250	0.83	27.3	15.5	4	232.3	131.0
A10	667.2	250	0.83	27.3	24.5	4	232.3	155.0

**Table 3:** Laser parameters used in the processing of sample shown in Figure 11, used for the EBSD studies. The sample is moving in the direction perpendicular to the laser beam scanning one at a velocity given by *v*_s_.

Frequency (kHz)	*F*_av_ (mJ/cm^2^)	*v*_L_ (mm/s)	<*F*_1D_> (J/cm^2^)	*l_L_* (mm)	*v*_s_ (mm/s)	<*F*_2D_> (J/cm^2^)
800	50	150	7.1	12	0.045	55.1

## Data Availability

Not applicable.

## Supplementary Materials

The following supporting information can be downloaded at: https://www.mdpi.com/article/10.3390/nano12142380/s1, Figure S1. EDS line profiles showing the increment on the W content from the external region of the top part of a nano-protrusion of A3 type to its interior. Figure S2. EDS line profiles in the regions between nano-protrusions of A3 type. Figure S3. Scheme of the crystallographic projections used to build Figure 11. RD and TD define the plane of the tape surface, ND is the normal direction.
