# Supplementary material for: Highly Regular Hexagonally-Arranged Nanostructures on Ni-W Alloy Tapes upon Irradiation with Ultrashort UV Laser Pulses
Source: Nanomaterials (Basel). 2022 Jul 12;12(14):2380. doi: 10.3390/nano12142380 (PMC9323319; doi:10.3390/nano12142380)
Supplement: Supplementary file 1 [file nanomaterials-12-02380-s001.zip › nanomaterials-1794877-supplementary.pdf]

nanomaterials

MDPI

Supplementary Materials

Highly regular hexagonally-arranged nanostructures on Ni-W alloy tapes upon irradiation with ultrashort UV laser pulses

Luis Porta-Velilla [1], Neslihan Turan [1,2], Álvaro Cubero [1], Wei Shao [1,3], Hongtao Li [1,4], Germán F. de la Fuente [1], Elena Martínez [1], Ángel Larrea [1], Miguel Castro [1], Haluk Koralay [2], Şükrü Çavdar [2], Jörn Bonse [5] and Luis A. Angurel [1,*]

[1] Instituto de Nanociencia y Materiales de Aragón (CSIC-University of Zaragoza), C/María de Luna 3, 50018 Zaragoza, Spain; porveli@unizar.es (L.P.-V.); neslihanturan@gazi.edu.tr (N.T.); acubero@unizar.es (Á.C.); wshao@bjut.edu.cn (W.S.); li_ht@163.com (H.L.); german.delafuente.leis@csic.es (G.F.d.l.F.); elenamar@unizar.es (E.M.); alarrea@unizar.es (Á.L.); mcastro@unizar.es (M.C.)

[2] Department of Physics, Faculty of Science, Gazi University, 06500 Teknikokullar Ankara, Turkey; koralay@gazi.edu.tr (H.K.); cavdar@gazi.edu.tr (Ş.Ç.)

[3] Institute of Welding and Surface Engineering Technology, Faculty of Materials and Manufacturing, Beijing University of Technology, Beijing 100124, China

[4] College of Materials Science and Engineering, Nanjing Tech University, Nanjing 210009, China

[5] Bundesanstalt für Materialforschung und -prüfung (BAM), Unter den Eichen 87, 12205 Berlin, Germany; joern.bonse@bam.de

* Correspondence: angurel@unizar.es

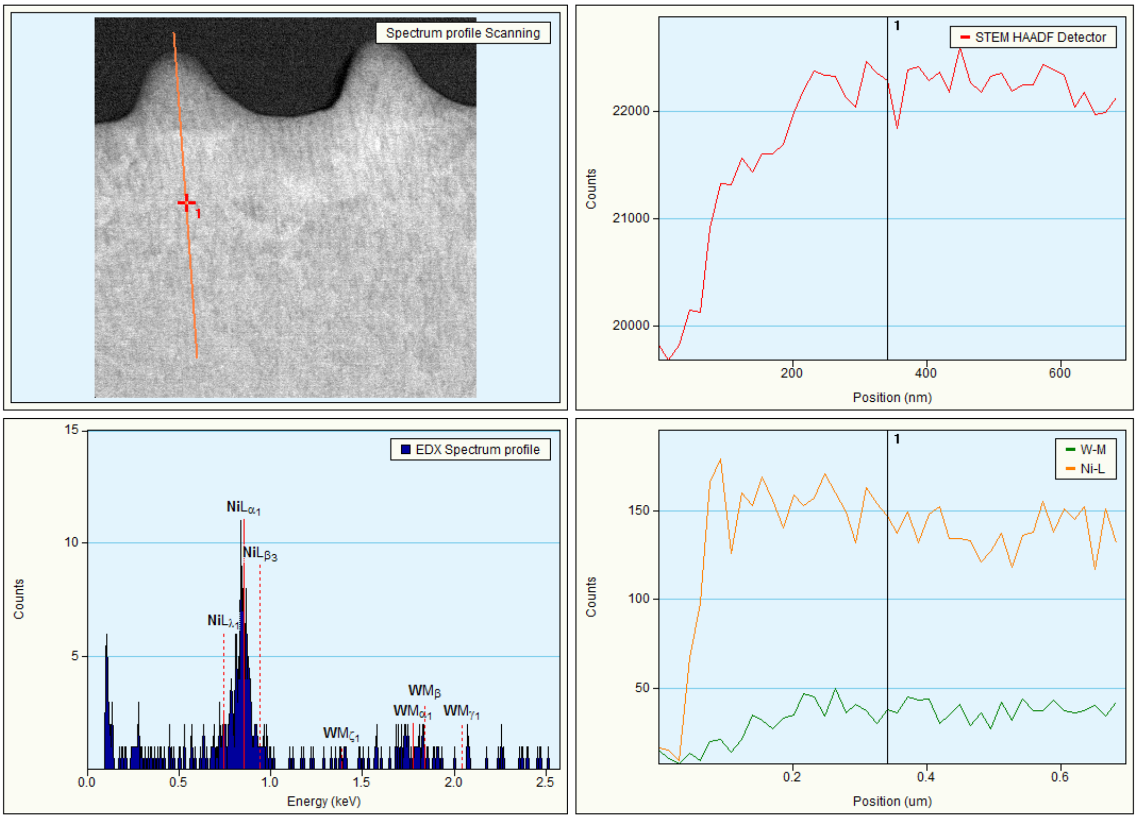

Figure S1. EDS line profiles showing that the W content increases from the top part of a nanoprotrusion of A3 type. The label "W-M" denotes the signal integrated over the tungsten W-Mα^1 line at 1.77 keV, while the label "Ni-L" denotes the signal integrated over the nickel Ni-Lα^1 line at 0.85 keV shown in the lower part of the figure.

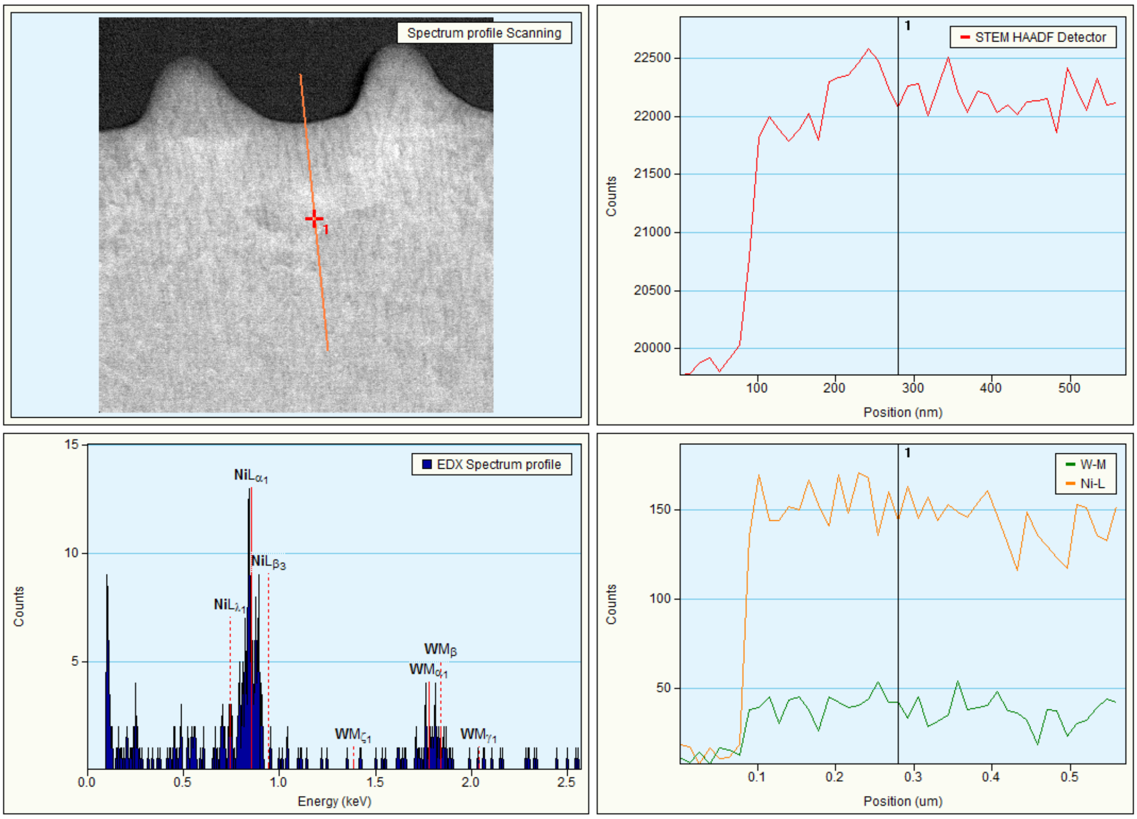

Figure S2. EDS line profiles in the regions between nanoprotrusions of A3 type.

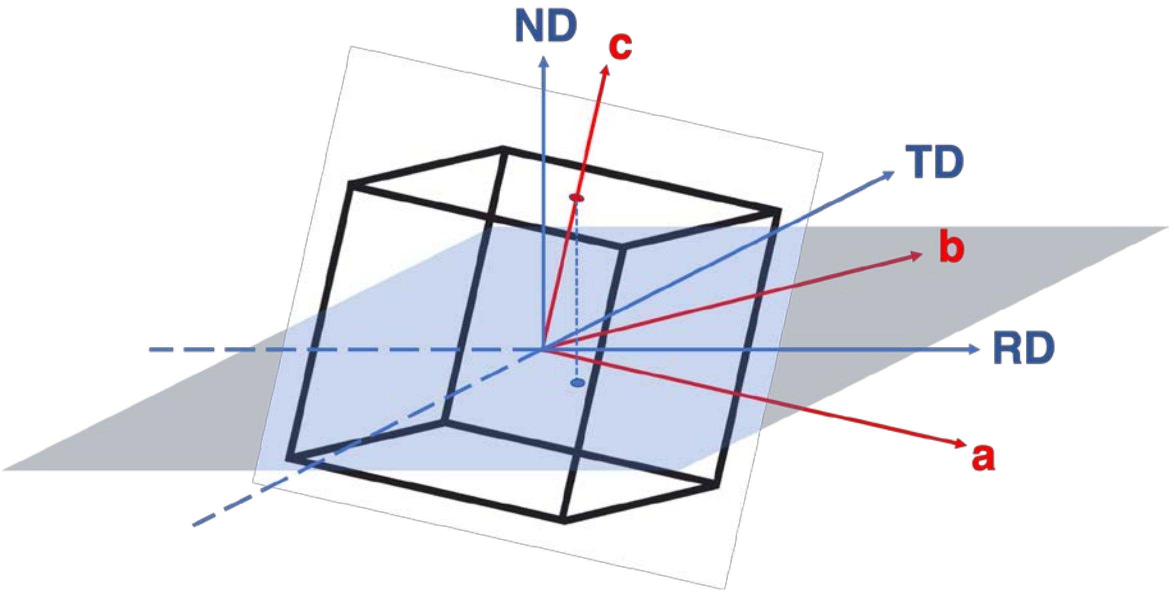

Figure S3. Scheme of the crystallographic projections used to build Figure 11. RD and TD define the plane of the tape surface, ND is the normal direction. The cube shows the crystallographic grain orientation with the particular a, b and c directions. Taking the refence origin at the center of the cube, the points indicated in Figure 11 are the projections over the tape plane of the center of the upper side of the cube. The blue point indicated in this scheme is one of the projected points encoded in Figure 11 for a specific grain.
